# Supplementary material for: The embryonic transcriptome of Parhyale hawaiensis reveals different dynamics of microRNAs and mRNAs during the maternal-zygotic transition
Source: Sci Rep. 2022 Jan 7;12:174. doi: 10.1038/s41598-021-03642-9 (PMC8741983; doi:10.1038/s41598-021-03642-9)
Supplement: Supplementary file 1 — Supplementary Information 1. [file 41598_2021_3642_MOESM1_ESM.pdf]

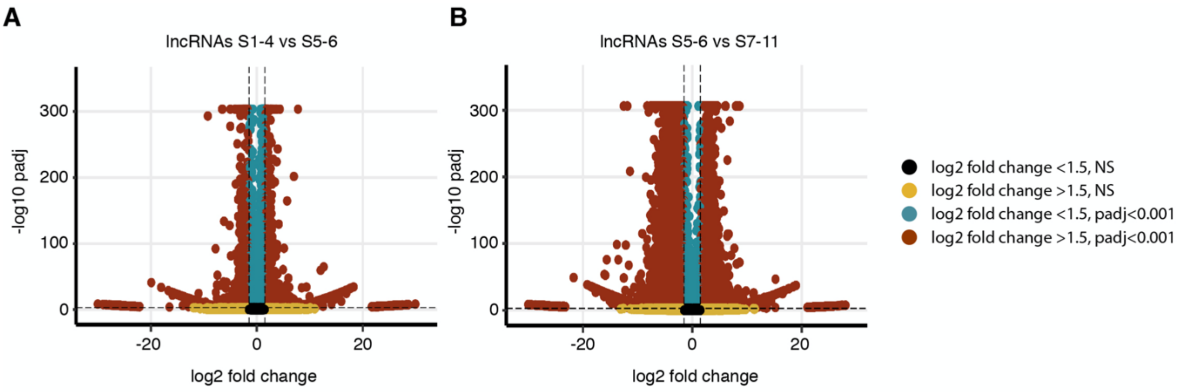

Supplementary figure 1. Differential expression analysis of lncRNAs during zygotic genome activation

(A and B) Volcano plots showing log2 fold change in expression (x-axis) versus the p-adjusted value (y- axis), for each lncRNA expressed between the first two time-points S1-4 to S6 **(A)** and S6 to S8-11 **(B)**. Only red dots (log2 fold change ≤ -1.5 or ≥ 1.5 with padj < 0.05) are considered significant.

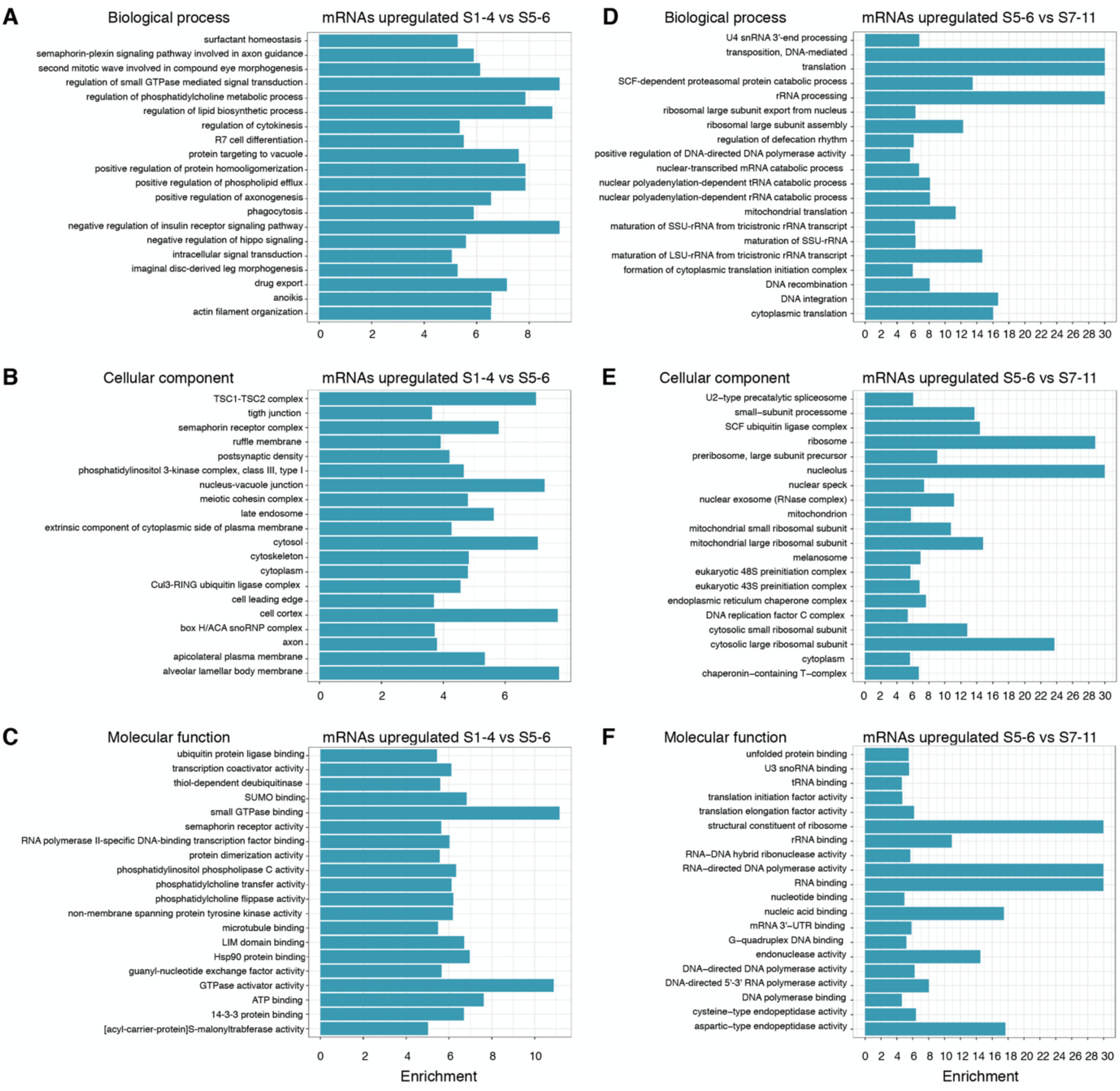

Supplementary figure 2. TopGO enrichment analysis results of upregulated mRNAs during the two waves of zygotic genome activation

Functional enrichment analysis was performed for the upregulated mRNAs between S1-4 and S6 **(A, B, C)** and between S6 and S8-S11 **(D, C, F)**. The top 20 enriched GO terms belonging to GO Biological Processes, GO Cellular Components, and GO Molecular Functions are shown for each comparison respectively.

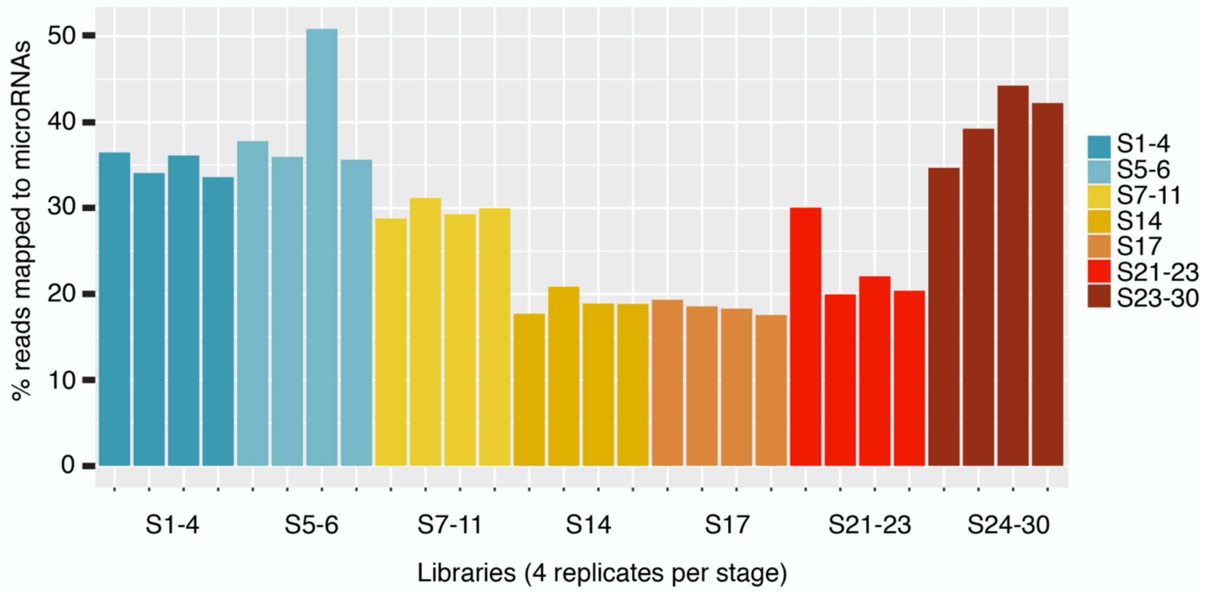

Supplementary figure 3. Percentage of reads mapped to annotated microRNAs for each sample sequenced.
