# Supplementary material for: The embryonic transcriptome of Parhyale hawaiensis reveals different dynamics of microRNAs and mRNAs during the maternal-zygotic transition
Source: Sci Rep. 2022 Jan 7;12:174. doi: 10.1038/s41598-021-03642-9 (PMC8741983; doi:10.1038/s41598-021-03642-9)
Supplement: Supplementary file 2 — Supplementary Information 2. [file 41598_2021_3642_MOESM2_ESM.pdf]

Table 1: Crustacean genomes from NCBI

**Specie**	**NCBI Assembly**
Hyalella azteca	GCA_000764305.2 Hazt_2.0
Triops cancriformis	GCA_000981345.1 tcf_1.0
Caridina multidentata	GCA_002091895.1 Cmul_gen_Assembly01
Ligia exotica	GCA_002091915.1 Lexo_gen_Assembly01
Penaeus japonicus	GCA_002291165.1 Mjap_WGS_v1
Procambarus virginalis	GCA_002838885.1 Pvir0.4
Eulimnadia texana	GCA_002872375.1 clam_shrimp_assembly_v0.1
Eriocheir sinensis	GCA_013436485.1 ASM1343648v1
Lepidurus apus	GCA_003723985.1 Lubb2018
Lepidurus arcticus	GCA_003724045.1 ASM372404v1
Penaeus vannamei	GCA_003789085.1 ASM378908v1
Daphnia magna	GCA_003990815.1 ASM399081v1
Palaemon carinicauda	GCA_004011675.1 ASM401167v1
Armadillidium vulgare	GCA_004104545.1 Arma_vul_BF2787
Pandalus platyceros	GCA_005815305.1 GSC_Sprawn_1.0
Trinorchestia longiramus	GCA_006783055.1 ASM678305v1
Tigriopus californicus	GCA_007210705.1 Tcal_SD_v2.1
Penaeus monodon	GCA_015228065.1 NSTDA_Pmon_1
Portunus trituberculatus	GCA_008373055.1 ASM837305v1
Armadillidium nasatum	GCA_009176605.1 CNRS_Arma_nasa_1.0
Cherax quadricarinatus	GCA_009761615.1 DU_Cquad_1.0
Amphibalanus amphitrite	GCA_009805615.1 SNU_Aamp_1
Cherax destructor	GCA_009830355.1 DU_Cdes_1.0
Pollicipes pollicipes	GCA_011947565.2 Ppol_2
Tigriopus kingsejongensis	GCA_012959195.1 ASM1295919v1
Caligus rogercresseyi	GCA_013387185.1 ASM1338718v1
Daphnia dubia	GCA_013387435.1 dubia_v0.01
Platorchestia sp	GCA_014220935.1 ASM1422093v1
Semibalanus balanoides	GCA_014673585.1 Sbal3.1
Orchestia grillus	GCA_014899125.1 Ogril_1
Macrobrachium nipponense	GCA_015104395.1 ASM1510439v1
Daphnia pulex	GCA_900092285.2 PA42 4.1
Oithona nana	GCA_900157175.1 O_NANA_1
Acartia tonsa	GCA_900241095.1 Aton1.0
Apocyclops royi	GCA_900607525.1 AroyWGS1.0
Tisbe holothuriae	GCA_900659605.1 ASM90065960v1
Eurytemora affinis	GCF_000591075.1 Eaff_2.0

Table 2: De novo transcriptome annotation statistics

**Transcriptome statistics**	
Total trinity genes	31087
Total trinity transcripts	49532
Percent GC	46.46
Contig N10	10065
Contig N20	7591
Contig N30	6090
Contig N40	4918
Contig N50	3934
Average length (bp)	2146.44
Median length (bp)	1211.5
Total assembled bases	106317470
**3'UTRs statistics**	
transcripts with 3'UTRs	42505
Average length (bp)	1135.70
Median length (bp)	319
Max length (bp)	22970
Min length (bp)	1
Sequences >10nt	30427
